# Evaluation of the World Health Organization—International Committee of the Red Cross Basic Emergency Care course for senior medical students

**DOI:** 10.1186/s12245-023-00487-z

**Published:** 2023-04-21

**Authors:** Nichole Michaeli, Giovanna De Luca, Mary Gitau, Justin Myers, Daniel Ojuka, Derick Ouma, Travis Wieland, Grace Wanjiku

**Affiliations:** 1 Department of Emergency Medicine, Warren Alpert Medical School of Brown University, 55 Claverick St., Providence, RI 02903 USA; 2 P.C.E.A. Chogoria Hospital, Chogoria, Kenya; 3 Department of Emergency Medicine, University of North Carolina at Chapel Hill, Chapel Hill, NC USA; 4 Department of Surgery, University Of Nairobi, College of Health Science, Nairobi, Kenya; 5 Department of Emergency Medicine, University of Wisconsin, Madison, WI USA

**Keywords:** Emergency medicine education, Training, Emergency care

## Abstract

**Background:**

The *Basic Emergency Care: Approach to the acutely ill and injured* course was developed to train health care providers to recognize, stabilize, and treat critically ill patients in resource-limited settings. This study evaluates the Basic Emergency Care course as a tool for improving the emergency medicine knowledge and skills of medical students in a lower-middle income country.

**Methods:**

This prospective study was conducted with senior medical students at the University of Nairobi School of Medicine in October 2021. Participants’ knowledge was assessed with multiple choice pre- and post-course examinations. Pre- and post-course surveys assessed participants’ confidence in managing acutely ill and injured patients using a 4-point Likert scale.

**Results:**

A total of 30 students from the graduating medical school class participated in the study. Post-course examination scores (mean 94.5%, range 80–100%) showed a significant improvement (*p* < 0.05) compared to pre-course examination scores (mean 82%, range 64–96%). Participants’ comfort and confidence in providing emergency care and performing critical emergency skills significantly increased (*p* < 0.05) between the pre- and post-course surveys.

**Conclusion:**

These findings suggest that the Basic Emergency Care course is effective in providing senior medical students with basic emergency medicine knowledge and increasing their confidence to identify and address life-threatening conditions prior to their intern year.

## Background

Emergency medical conditions are responsible for over 28 million deaths annually with low- and middle-income countries (LMICs) bearing a disproportionate burden of morbidity and mortality [1–3]. Access to high quality emergency care has the potential to reduce this disparity, but many LMICs lack organized emergency medical systems and dedicated emergency medicine (EM) trained providers [3–5]. Specialized EM training programs are in the early stages of development in many LMICs, and emergency departments are often staffed by physicians who are not EM-trained [6, 7]. This contributes to shortcomings in triage and the early recognition and treatment of critically ill patients.

Many programs ranging from short interventions to full residency programs have been developed to train healthcare providers from LMICs in the delivery of emergency care [7]. The development of EM residency programs requires significant financial, political, and human resources [8–10]. However, short-duration EM courses offer a reasonable alternative to increase clinical knowledge, competency, and procedural skills of emergency care workers [11–14]. Both EM residency programs and short-duration EM interventions are associated with significant reductions in mortality [15, 16].

As EM residency programs are slow to expand in LMICs, short-duration EM training courses can provide a feasible, efficient, and cost-effective means to bridge the gap and provide health care workers with critical EM knowledge and skills. The Basic Emergency Care (BEC) course was developed by the World Health Organization (WHO) in collaboration with the International Committee of the Red Cross (ICRC) and the International Federation for Emergency Medicine (IFEM) to train health care providers to manage acute illness and injury in resource-limited settings [17]. This open-access, 4 to 5-day course utilizes lectures, small group discussions, and skills stations to teach all levels of health care providers to recognize, stabilize, and manage critically ill and injured patients. It has been successfully piloted among clinical officers, assistant medical officers, medical officers, nurses, and nursing assistants in several LMICs [18, 19].

This study aims to evaluate the BEC course as a tool for improving the emergency medicine knowledge and skills of senior medical students in Kenya, a lower-middle income country. EM training courses in several LMICs have focused on medical students in an effort to increase the number of providers with EM knowledge and improve access to emergency medical care [20–22]. Kenyan medical students represent a unique target population because there is limited dedicated EM training in the medical school curriculum, and the majority of Kenyan physicians will work as general practitioners after completing a one-year internship [14, 23]. Therefore, offering the BEC course to senior medical students can potentially increase exposure to EM concepts and skills and expand the number of future physicians with EM training.

The first medical school in Kenya was established at the University of Nairobi in 1967 [24]. Undergraduate medical education consists of six years divided between pre-clinical courses and clinical rotations [14, 23]. Kenyan medical students have the opportunity to participate in a 2-day trauma course during their 6th year, but there are no other dedicated emergency medicine courses or rotations in the medical school curriculum [14]. After graduation, the new physicians complete 12-month internships at district hospitals throughout the country and rotate through internal medicine, surgery, pediatrics, obstetrics/gynecology, psychiatry, and community health departments [23]. Following internship, some physicians will pursue advanced specialized training, in the form of certificate, diploma, and Masters of Medicine programs, but the majority of Kenya physicians will work as general practitioners [23].

As the Kenyan Ministry of Health aims to prioritize emergency medical care [23], expanding access to focused EM training opportunities is crucial. Although the BEC course has been successful in other settings [18, 19], this course has not previously been evaluated in Kenya or among senior medical students. We hope to show that the BEC course can provide senior medical students with the emergency medicine knowledge and skills needed to care for acutely ill and injured patients.

## Methods

This prospective study was conducted with senior medical students at the University of Nairobi School of Medicine in October 2021. A needs assessment was distributed to gauge confidence in managing emergency medicine cases and interest in participating in an emergency medicine training. Based on overwhelming interest in emergency medicine training, we proceeded with the BEC course.

Participants were recruited via an advertisement in the University of Nairobi School of Medicine Class of 2021 WhatsApp group [25]. Participants were selected on a continuous, first-come basis. All members of the graduating class at the University of Nairobi School of Medicine were eligible to participate. The course was capped at 30 participants according to African Federation for Emergency Medicine (AFEM) regulations [26].

The graduating medical students participated in the 5-day course which included lectures, small group discussions, and skills stations covering the ABCDE approach, trauma, difficulty in breathing, shock, and altered mental status. Participants’ knowledge was assessed with 25 question, multiple choice, pre- and post-course examinations. A pre- and post-course survey assessed participants’ confidence in managing acutely ill and injured patients using a 4-point Likert scale with responses ranging from 1 (strongly disagree) to 4 (strongly agree) and 1 (not confident at all) to 4 (very confident). The pre- and post- course surveys included both positive (i.e., I feel confident seeing very ill patients) and negative (i.e., I do not feel confident in my knowledge of emergency care) statements. The responses to the negative statements were not reversed to calculate the mean Likert scores. The pre- and post-course examinations and surveys were the standard versions developed as part of the BEC training package, which were obtained from the AFEM BEC coordinator [27]. These materials can be accessed upon request to AFEM [27].

De-identified participant data was prospectively collected during the course. Data were managed on a password-protected computer. Quantitative data from pre- and post-course examinations and surveys were compared using the paired two-sample *t*-test. All analysis was completed using the R statistics software [28].

## Results

### Demographics

A total of 30 students from the graduating class at the University of Nairobi School of Medicine participated in the BEC course. The mean age of the participants was 26.3 years, 63% were female, and 37% were male. All of the participants had recently completed medical school rotations at the time of the study, but they had not started their post-medical school internship. None of the participants had prior emergency medicine training or experience with the BEC course.

### Pre- and post-tests

The pre- and post- course tests were completed by 30 participants. Post-course examination scores (mean 94.5, range 80–100) showed a significant improvement (*p* < 0.05) compared to pre-course examination scores (mean 82, range 64–96) (Table 1).

**Table 1:** Pre- and post-course test scores

Pre-test mean (range) *n* = 30	Post-test mean (range) *n* = 30	Difference
82 (64–96)	94.5 (80–100)	*p* < 0.05

### Pre- and post-course surveys

The pre- and post- course tests were completed by 30 participants. However, three participants did not answer some of the pre-course survey questions and one participant excluded a question on the post-course survey. To account for participants who did not answer questions on the pre- and post-course surveys, we excluded those participants from the pre-/post-survey pairing for those specific questions, while including their responses in the analysis for all other questions.

There was a significant increase (*p* < 0.05) in self-reported comfort and confidence in providing emergency care and performing critical emergency skills between the pre- and post-course surveys (Table 2). In addition, participants unanimously “strongly agree” that emergency care training for health care providers is important.

**Table 2:** Pre- and post-course survey results

		**Pre-Course Survey**		**Post-Course Survey**		
	**Question**	***N***	**Mean (SD)**	***N***	**Mean (SD)**	**Paired T-Test**
**1 (strongly disagree) to 4 (strongly agree)**	I feel comfortable handling any patient requiring emergency care	30	2.27 (0.52)	30	3.77 (0.43)	*p* < 0.001
	I feel nervous about seeing patients with emergencies	30	3.1 (0.61)	30	1.97 (0.85)	*p* < 0.001
	I feel that others in my clinical unit have the knowledge and skills to handle emergency care patients	30	2.73 (0.64)	30	3.17 (0.7)	*p* = 0.021
	I feel that I lack the skills to provide care in most emergencies	29	2.52 (0.69)	30	1.33 (0.55)	*p* < 0.001
	I feel prepared to see emergency care patients in my clinical setting	29	2.28 (0.59)	30	3.47 (0.51)	*p* < 0.001
	I feel confident seeing very ill patients	29	2.24 (0.58)	29	3.38 (0.49)	*p* < 0.001
	I feel uncomfortable using standard emergency protocols	30	2.33 (0.66)	30	1.4 (0.67)	*p* < 0.001
	I feel that I understand the ABCDE’s of basic emergency care	29	3.1 (0.72)	30	3.83 (0.38)	*p* < 0.001
	I feel I have an organized approach that allows me to be prepared for all emergency care patients	30	2.37 (0.72)	30	3.8 (0.41)	*p* < 0.001
	I do not feel confident in my knowledge of emergency care	30	2.53 (0.73)	30	1.27 (0.45)	*p* < 0.001
	Emergency care trainings for generalist healthcare providers are important	30	4 (0)	30	4 (0)	*p* = 0.326
**1 (not confident at all) to 4 (very confident)**	Emergency management of the acutely ill adult	29	2.14 (0.58)	30	3.43 (0.5)	*p* < 0.001
	Emergency management of the acutely ill child	29	2 (0.71)	30	3.43 (0.57)	*p* < 0.001
	Emergency management of the injured adult	29	2.07 (0.65)	30	3.6 (0.5)	*p* < 0.001
	Emergency management of the injured child	29	1.79 (0.68)	30	3.47 (0.57)	*p* < 0.001
	Emergency management of the patient with Shock	29	2.17 (0.71)	30	3.63 (0.49)	*p* < 0.001
	Emergency management of the patient with altered mental state	29	1.83 (0.71)	30	3.53 (0.63)	*p* < 0.001
	Emergency management of the patient with difficulty in breathing	29	2.17 (0.66)	30	3.67 (0.48)	*p* < 0.001
	Understanding of emergency drugs	29	1.76 (0.83)	30	3.37 (0.56)	*p* < 0.001
	Have skills to manage an obstructed (blocked) airway	29	1.76 (0.64)	30	3.6 (0.5)	*p* < 0.001
	Have skills to manage a patient with difficulty in breathing	29	2.07 (0.65)	30	3.63 (0.49)	*p* < 0.001
	Have skills to manage a patient with bleeding problems	29	2 (0.65)	30	3.6 (0.5)	*p* < 0.001
	Have skills to immobilize patients	29	1.72 (0.7)	30	3.77 (0.43)	*p* < 0.001

## Discussion

The results from the pre- and post-course examinations suggest that the BEC course is effective in improving senior medical students’ emergency medicine knowledge and skills. The pre-course examination scores (82, range 64–96) were quite high, which implies that students had a solid foundation of emergency medicine knowledge prior to taking the BEC course. However, the statistically significant increase in post-course examination scores (94.5, range 80–100) represents an overall increase in knowledge following the course. The pre- and post-course surveys also show that the BEC course is effective in increasing graduating medical students’ confidence in managing critically ill and injured patients. The increase in the students’ confidence in managing patients with emergencies may indicate the usefulness of the teaching strategies employed by the course including an algorithmic approach to emergency care and opportunities for hands-on skill practice.

The BEC course has been successfully adopted by pre-hospital and hospital-based providers in multiple LMICs, but this is the first time that the BEC course was specifically taught to graduating medical students, to our knowledge. Our findings indicate that the BEC course is a useful means to provide senior medical students in Kenya with basic emergency medicine knowledge and a systematic approach to identifying and addressing life-threatening conditions prior to their intern year. This training may have the added benefit of furthering the development of EM as a specialty, as more medical students become familiar with EM knowledge and potentially elect to pursue EM residency or diploma programs.

Scheduling the BEC course at the end of the medical school term was an optimal time to conduct the training. The participants had completed their medical school studies and clinical rotations, but most students were still living in Nairobi awaiting graduation. This allowed us to reach a large number of students, while not interfering with medical school activities. It would be logistically difficult to conduct a 5-day training during the medical school term, as the students have limited time away from medical school classes and rotations. Efforts to incorporate this training into the medical school curriculum will involve administrators and key stakeholders at the medical school.

While the usefulness and effectiveness of this course were demonstrated in this study, it is important to address the feasibility of replicating this course, especially in LMICs. AFEM regulations impact the number of participants that can be trained at each BEC course. Therefore, this course was capped at 30 participants [25]. Since each medical school class at the University of Nairobi has approximately 250 students, multiple trainings would be necessary to provide the course to all students.

The total cost of the 5-day course was approximately 5000 USD, which included the cost of the venue, refreshments for participants, supplies, and printing costs. This does not include the cost of flights and accommodations for international trainers which separately totaled approximately 4000 USD. When only taking into account the venue, refreshments, supplies, and printing costs, the average cost per participant is approximately 160 USD. However, if you include the cost of flights and accommodations for visiting trainers, the average cost per participant is nearly double at 300 USD.

While many of the supplies can be reused for future courses, the venue, refreshments, and printing costs (approximately 3500 USD) will accrue with each course. Steps to reduce the total cost of the course should be considered to make this course more financially feasible to groups with limited funding. Future facilitators could consider requesting donation of venue space from local universities or religious groups. Facilitators should also make conscious efforts to use locally available materials for skills stations. Participants could also be asked to contribute a small fee to attend the course, although this may cause the course to become cost-prohibitive to some. Alternatively, medical schools could allocate tuition funding towards the course.

The best way to reduce the overall cost of the course, however, involves training more local health care providers. This BEC course was conducted through a collaboration with Kenyan and international physicians. In accordance with WHO and AFEM regulations, course facilitators must have completed both the BEC and Training of Trainers (ToT) course as participants prior to teaching the course [25]. In addition, BEC courses must be conducted under the supervision of a Master Trainer, a facilitator who has both completed the BEC and ToT courses as a participant and has taught BEC courses under another Master Trainer [25]. Given the country or region in which the BEC course is conducted, there may not be local Master Trainers available. This was the first BEC course held in Kenya, and there were no Kenyan Master Trainers at the time of the study. An international Master Trainer was able to join the course, but planning for a Master Trainer, particularly in areas where the BEC is not established, is an important consideration. As more BEC courses are conducted in the same location, particularly in conjunction with the BEC Training of Trainers course, local institutions will be less reliant on visiting international Master Trainers and facilitators.

## Limitations

While the BEC course was developed to have universal applicability, this study represents the first implementation of this course in Kenya. It was also conducted at a single site, the University of Nairobi School of Medicine. Further work is needed to expand the BEC course to medical schools throughout Kenya and other LMICs to better determine the generalizability of this specific approach to education in basic emergency care.

This study showed short-term increases in basic emergency medicine knowledge and confidence in managing critically ill patients. Further studies are necessary to evaluate long-term retention of knowledge and skills. In addition, this study did not evaluate the clinical impact of the BEC course. While this would be logistically difficult with our study population, since they are completing their intern year at different sites, this might be more feasible with courses conducted for providers at a single site.

## Conclusions

These findings suggest that the BEC course is effective in providing senior medical students with basic emergency medicine knowledge and increasing their confidence to identify and address life-threatening conditions prior to their intern year. Introducing the BEC course at the medical school level may offer a valuable means to increase emergency medicine training in Kenya. Future work should determine if the BEC course is appropriate for medical students in other LMICs.

## Data Availability

All datasets used and/or analyzed during the current study are available from the corresponding author on reasonable request.

## Abbreviations

AFEM: African Federation of Emergency Medicine
BEC: Basic Emergency Care: Approach to the acutely ill and injured Course
EM: Emergency medicine
HICs: High-income countries
ICRC: International Committee of the Red Cross
IFEM: International Federation for Emergency Medicine
LMICs: Low- and middle-income countries
ToT: Training of Trainers
WHO: World Health Organization
